# Optimized full-spectrum flow cytometry panel for deep immunophenotyping of murine lungs

**DOI:** 10.1016/j.crmeth.2024.100885

**Published:** 2024-10-30

**Authors:** Zora Baumann, Carsten Wiethe, Cinja M. Vecchi, Veronica Richina, Telma Lopes, Mohamed Bentires-Alj

**Affiliations:** 1 Department of Biomedicine, University Hospital Basel, University of Basel, 4031 Basel, Switzerland; 2 Department of Surgery, University Hospital Basel, 4031 Basel, Switzerland; 3 BioLegend, Inc., San Diego, CA 92121, USA

**Keywords:** full-spectrum flow cytometry, high-dimensional single-cell analysis, murine lung immunophenotyping, cancer immunology, metastasis, immunophenotyping, immunology, breast cancer, oncology

## Abstract

The lung immune system consists of both resident and circulating immune cells that communicate intricately. The immune system is activated by exposure to bacteria and viruses, when cancer initiates in the lung (primary lung cancer), or when metastases of other cancer types, including breast cancer, spread to and develop in the lung (secondary lung cancer). Thus, in these pathological situations, a comprehensive and quantitative assessment of changes in the lung immune system is of paramount importance for understanding mechanisms of infectious diseases, lung cancer, and metastasis but also for developing efficacious treatments. Unfortunately, lung tissue exhibits high autofluorescence, and this high background signal makes high-parameter flow cytometry analysis complicated. Here, we provide an optimized 30-parameter antibody panel for the analysis of all major immune cell types and states in normal and metastatic murine lungs using spectral flow cytometry.

## Highlights

• Generation of a murine lung immunophenotyping panel using spectral flow cytometry
• This panel enables concurrent analysis of 17 major lung immune cell types and states
• Autofluorescence segmentation decreases unmixing noise
• Unsupervised clustering helps identify immune cell subsets in metastatic lungs

## Motivation

Full-spectrum flow cytometry permits the use of more complex and high-parameter antibody panels compared to conventional techniques. However, the combination of many antibodies may result in unmixing issues that lead to inaccurate data unless the panels are established and optimized thoroughly. In addition, tissue autofluorescence (e.g., in lung) adds noise to the signal, which can impact the resolution of immunophenotyping panels. This challenge can be overcome by defining and segmenting organ autofluorescence signatures. Here, we report a ready-to-apply optimized high-parameter antibody panel for lung immunophenotyping.

## Graphical abstract

**Figure:**
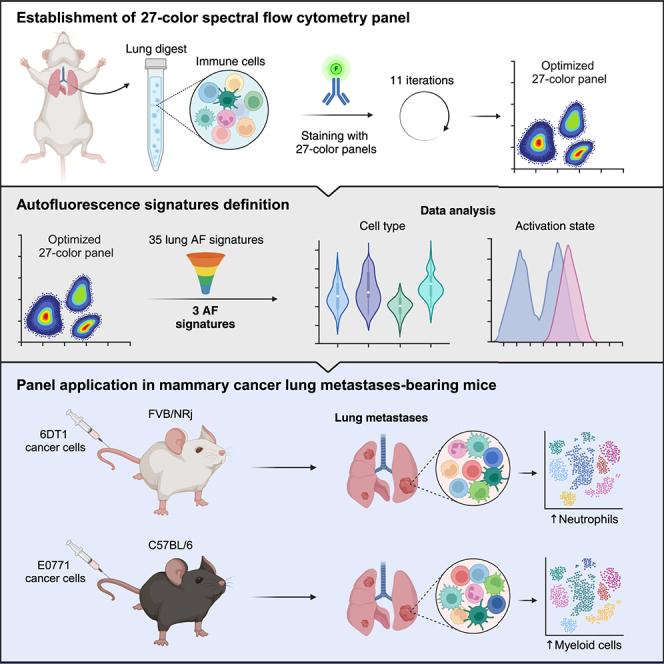

## Teaser

Baumann et al. have compiled, tested, and optimized a 30-parameter murine lung immunophenotyping panel. This allows concurrent analysis of all major immune cell types and states. Furthermore, autofluorescence signatures were established, and the antibody panel was applied in two mammary cancer lung metastasis models.

## Introduction

The use of *Mus musculus* as an animal model to gain insight into dynamic cell-cell interactions in both healthy and disease states requires a comprehensive characterization of its immune cell types. To date, we have lacked a panel with an extensive pool of antibodies that recognize cell-surface proteins and would permit broad immunophenotyping in a highly autofluorescent organ like the lung.

Breast cancer affects one in eight women during their lives.^1^ Metastases are the fatal hallmark of the disease, occurring most frequently in the lung, brain, bone, liver, and lymph nodes.^2^^,^^3^ Whereas survival of patients with early-stage breast cancer has improved in the last two decades, the survival of late-stage metastatic patients remains limited.^4^ The immune system has been at the forefront of oncology drug discovery, with therapies such as immune checkpoint blockade already approved for some cancer types. Yet, resistance to such treatments occurs, and benefits remain limited for many patients. Therefore, in-depth preclinical studies are needed to improve our understanding of the metastatic immune tumor microenvironment.

As the immune system in metastasis is very heterogeneous and continuously evolves, it is of utmost importance to monitor immune cell populations and states throughout the metastatic process, from the formation of the pre-metastatic niche to overt lung metastases.^5^ Conventional flow cytometry is commonly used to assess the tumor immune microenvironment. However, it is limited by the number of fluorochromes that can be simultaneously analyzed. This often limits the complexity of sample analysis and, thus, increases the workload by requiring multiple panels for various cell types. Spectral flow cytometry addresses these issues by analyzing the full emission spectra across all lasers, allowing greater flexibility in panel design and size.^6^ Even though this technique is becoming more widely available, highly multiplexed phenotyping panels still require thorough optimization, especially when characterizing more complex and heterogeneous tissues like the lung.

Most published studies focus only on specific lung immune cells and lack overarching immune cell subtype identification. Recently, one study described a 13-parameter backbone panel to characterize normal and cancerous tissues, including the lung.^7^ This panel can easily be expanded to at least 21 parameters without redesign. Another study investigated leukocyte trafficking in the lung during infections using intravascular and intratracheal immune cell labeling of leukocytes, followed by a 20-parameter staining.^8^ The study included lineage markers for population identification but no activation or inhibitory markers. These reports emphasize the difficulty in establishing high-parameter lung panels. We have now established a 27-color (30-parameter, including three autofluorescence [AF] signatures) full-spectrum flow cytometry antibody panel to profoundly characterize the major immune cell subsets and states in the murine lung. The optimized panel described here was designed for immunophenotyping naive murine lungs and mammary cancer lung metastases.

Notably, this panel can be applied not only to metastases in the lung, but also to other lung diseases like viral infections, asthma, or chronic obstructive pulmonary disease (COPD). In-depth characterization of murine innate and adaptive immune cell populations using this flow cytometry panel will help reveal complex and dynamic immune responses on a high-dimensional single-cell level in preclinical models of human disease.

## Results

### Marker selection

We aimed to build a high-parameter panel (see STAR Methods and Table S1) to immunophenotype mice before and during the development of mammary lung metastases and to assess evolving changes in immune cell populations and their activation or inhibition.

**Figure 1:**
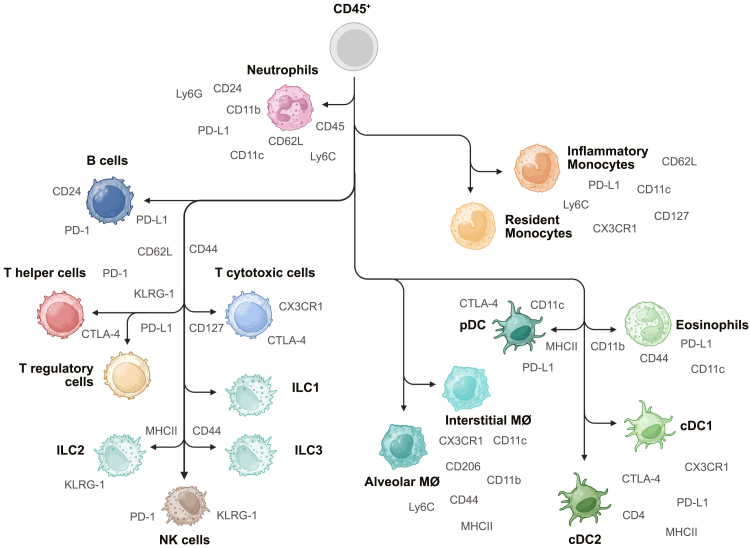
Immune subpopulations identification schematic Schematic of immune cell subpopulation identification. Possible markers for analysis are marked in dark gray. This is an examplary list and can be adapted to the needs of the user. ILC, innate lymphoid cells; MØ, macrophages, cDC, conventional dendritic cells; pDC, plasmacytoid dendritic cells.

For the initial marker selection, we aimed to identify 17 major immune cell populations in the lung (Figure 1). In the myeloid compartment, we included neutrophils, inflammatory and resident monocytes, eosinophils, conventional dendritic cells (cDCs) 1 and 2, and plasmacytoid DCs (pDCs), as well as alveolar (AMØs) and interstitial macrophages (IMØs). For the lymphoid lineage, we sought to identify B cells, T helper cells, regulatory T cells, cytotoxic T cells, natural killer (NK) cells, and innate lymphoid cells (ILCs) 1–3. For all cell types, we aimed to minimize the number of markers needed for population identification (Table S1). To achieve this, we based our approach on published lung immunophenotyping strategies.^9^^,^^10^ Besides our aim to minimize the number of lineage markers, we also focused on further sub-differentiating markers expressed by multiple cell populations. Therefore, we selected CD103 and CD11b for classifying cDC1 and -2 instead of XCR1 and SIRPα. Both options are commonly used in the literature.^11^^,^^12^^,^^13^^,^^14^ To discriminate between AMØs and IMØs, we strategically used antibodies targeting CD64, CD11c, CD11b, CX3CR1, and CD206 as published previously.^10^ However, another possibility is to use Siglec F, which is expressed only on AMØs and eosinophils.^10^

To facilitate the implementation and simplification of the panel into pre-existing wet-lab workflows, we selected only surface receptors, avoiding the necessity for cell fixation and intracellular staining. For identifying regulatory T cells, we thus chose to identify them as CD4^+^CD25^+^CD127^low^ instead of the commonly used CD4^+^FoxP3^+^.^7^^,^^15^^,^^16^ Notably, the panel can also be used with sample fixation post-staining, providing greater flexibility regarding data acquisition.

To identify all lung cell types of interest, 18 antibodies and a viability dye for the lineage cocktail are needed. However, to comprehensively understand the co-evolution of the immune system during cancer progression, we included several cell characterization markers, such as immune checkpoint inhibitors CTLA-4, PD-1, and PD-L1. For analyzing the maturation of different cell types, we added CD62L, CD44, CX3CR1, and CD122. Additionally, many lineage markers used for identifying cell populations can also be analyzed on other cell subsets, such as CD127, CD11c, Ly6C, CD24, KLRG-1, major histocompatibility complex (MHC) class II, and CD45 (Figure 1).

The following paragraph highlights possible subset identifications with our marker selection. Within the myeloid compartment, mature (aged) neutrophils can be identified by high expression of CD11b, CD11c, CD24, and CD45, while immature (newly released from the bone marrow) neutrophils are marked by elevated CD62L and Ly6C levels.^17^ Moreover, PD-L1 expression is heightened in neutrophil-mediated immune suppression during lung metastases and upon viral infections.^18^^,^^19^^,^^20^ Monocytes and macrophages are very plastic immune cells.^21^ Inflammation-triggered monocytes exhibit lower Ly6C and higher CD11c and MHC class II expression, with CX3CR1 as a marker for general immune cell recruitment.^22^^,^^23^ Generally, the lung contains MHC class II^+^ monocyte populations that increase with age.^24^^,^^25^ Moreover, CD127 expression results in the functional heterogeneity of monocyte responses in inflammatory diseases, including viral lung infections and rheumatoid arthritis.^26^ In lung metastasis, tumor-associated macrophages can be analyzed using MHC class II, CD11b, and Ly6C.^9^ pDCs can be identified as CD24^−^CD64^−^CD11c^+^Ly6C^+^ and additionally as CD4^−/+^.^11^^,^^27^ Pre-DCs can be analyzed within the CD11b^−^ population as CD103^+^CD11c^hi^.^28^ Notable co-stimulatory markers on lung DCs are PD-L1 and CTLA-4, whose expression has tumor-promoting effects due to the lowering of immune cell infiltration.^29^

As mentioned, the panel includes a broad range of immune checkpoint molecules, whose expression is crucial for the success of cancer immune checkpoint therapies. PD-1 can limit antitumor immunity when bound by the PD-L1 expressed by cancer cells.^30^^,^^31^ CTLA-4 represses T cell proliferation and cytokine production, and a CTLA-4-blocking antibody is already used in the clinics.^32^ Importantly, immune checkpoint molecules are not solely expressed on T cells, and their assessment on other cell types, including macrophages, is of importance.^33^^,^^34^

Additional characterization of CD4^+^ and CD8^+^ effector T cells (T_EFFs_) can be achieved by CD127 and KLRG-1, which are associated with heightened cytotoxic activity.^16^^,^^35^ KLRG-1^low^CD127^high^ are memory precursor effector cells, and KLRG-1^high^CD127^low^ are short-lived effector cells.^35^ Antigen-experienced T cells can be identified as CX3CR1^+^. Notably, CX3CR1 expression shortly after immune checkpoint blockade is a positive predictive marker of response and survival in patients with non-small cell lung cancer.^36^ Gradient CX3CR1 expression was recently reported to mark differentiation states of human and murine T cells, thus enabling cross-species interpretation.^37^ B cells expressing PD-L1 are critical for humoral immunity, particularly as regulatory B cells expressing high PD-L1 levels are crucial for the expansion and differentiation of T follicular helper cells.^38^ Additionally, regulatory B cells expressing PD-L1 have been associated with a higher abundance of T regulatory cells (T_regs_) in invasive breast cancer.^39^ Thus, the use of 26 well-targeted antibodies and a viability dye can reveal the lung immune cell diversity, including during metastasis.

### Panel design and gating strategy

The stepwise development and optimization processes were influenced by the unique opportunity of spectral flow cytometry that (1) enables the choice of a broader set of fluorochrome-conjugated antibodies, even with similar peak emissions, and (2) defines AF signatures, which are very high in the lung.^8^ The following strategies were employed for the initial establishment of the panel.

First, the antigens were classified into primary, secondary, and tertiary categories based on their expression levels.^40^ Because of their low expression, tertiary antigens were assigned a fluorochrome first, followed by secondary and primary (Table S2). Several antibodies, especially those of tertiary epitopes (i.e., CD127 in BV605, CD122 in APC, and KLRG-1 in PE-Cy7), were maintained based on previous successful experiments (data not shown). Second, bright fluorochromes were chosen for low-expressed epitopes, and vice versa, to maximize sensitivity.^40^ We assigned primary antigens to dim fluorochromes, such as CD45 to Spark Blue 550 and CD4 to Pacific Blue (Table S2). Third, fluorochromes with high spectral overlap were not assigned to epitopes co-expressed on a cell subset (Figure S1).

**Figure 2:**
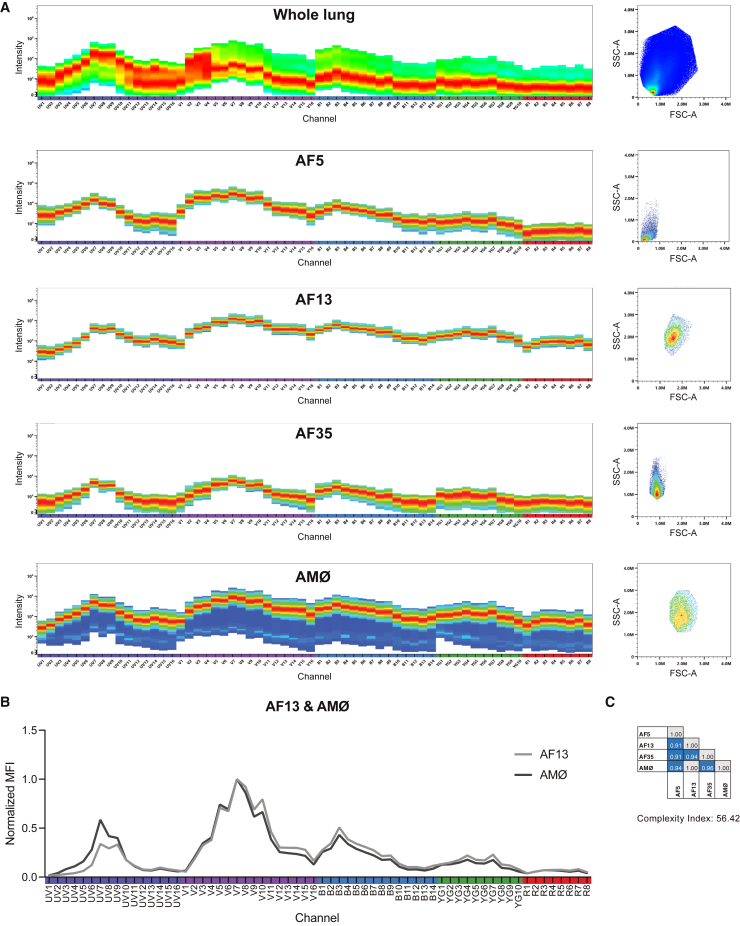
Lung AF signatures (A) Whole lung sample autofluorescence (AF; top) and the three unique AF signatures 5, 13, and 35 with their corresponding SSC-A and FSC-A values. Pure alveolar macrophage (AMØ) AF signature from bronchoalveolar lavage-isolated AMØs. (B) Normalized mean fluorescence intensity (MFI) showing the spectral overlap of AF13 with the AMØ signature over the 64 detectors. (C) Similarity indices for the individual AF signatures and the AMØs. Zero indicates no spectral overlap, and one denotes identical spectra.

The combination of spectrally similar fluorochromes requires analysis of the highly overlapping spectra. We used several approaches to set up, assess, and optimize the panel. This included analyzing similarity and complexity indices, conducting antibody titrations and stain index calculations, performing spread analysis, and comparing signal intensity in samples that were stained with one or with all antibodies.^15^^,^^41^ Antibody-fluorochrome conjugates were chosen based on high resolution and reduced spread in other channels. For unmixing the data, we used either beads or cells based on the one that yielded reduced spread and higher signal intensity. Through 11 iterations, we optimized the antibody-fluorochrome combinations. Notably, we left several brilliant ultra violet (BUV) and brilliant violet (BV) channels free, which correspond to peak areas of the lung AF spectrum (Figures 2A and S1A). In the final panel, fluorochrome pairs with similarity indices above 0.8 were present in two instances where the antigens were not co-expressed (Figure S1B).

The similarity indices from single-stained beads for unmixing do not account for the sample AF. To assess the accuracy of the unmixing result of the fully stained sample, data were gated on cells/single cells/live cells, and NxN permutation plots were inspected. We found that unmixing was accurate using beads for most antibodies. As the spectra of some fluorochromes differ when binding to cells or beads, NxN plots are essential for assessing unmixing accuracy.^15^ For most antibodies, especially in the UV and violet channels, the use of cells was less accurate than beads for unmixing, possibly because of cellular AF. In contrast, for PE-Fire810, APC, and AF700, the use of cells improved unmixing.

In summary, whereas following all standard guidelines for high-parameter panel design helps in predicting fluorochrome interactions, it does not consider cellular AF. Therefore, we tailored the antibody combinations to fit the lung AF profile (Figure 2A, top).

### Definition of AF signatures

Cellular AF arising from structural proteins (e.g., collagens or elastins), metabolites (e.g., NAD(P)H or tryptophan), and cellular organelles (e.g., lysosomes or mitochondria) can interfere with unmixing.^42^^,^^43^ For example, lung-resident AMØs are the most autofluorescent of all murine tissue-resident macrophages.^43^^,^^44^ This suggests that defining multiple AF signatures can facilitate unmixing beyond what could be achieved by shuffling antibody-fluorochrome conjugates within the panel.

**Figure 3:**
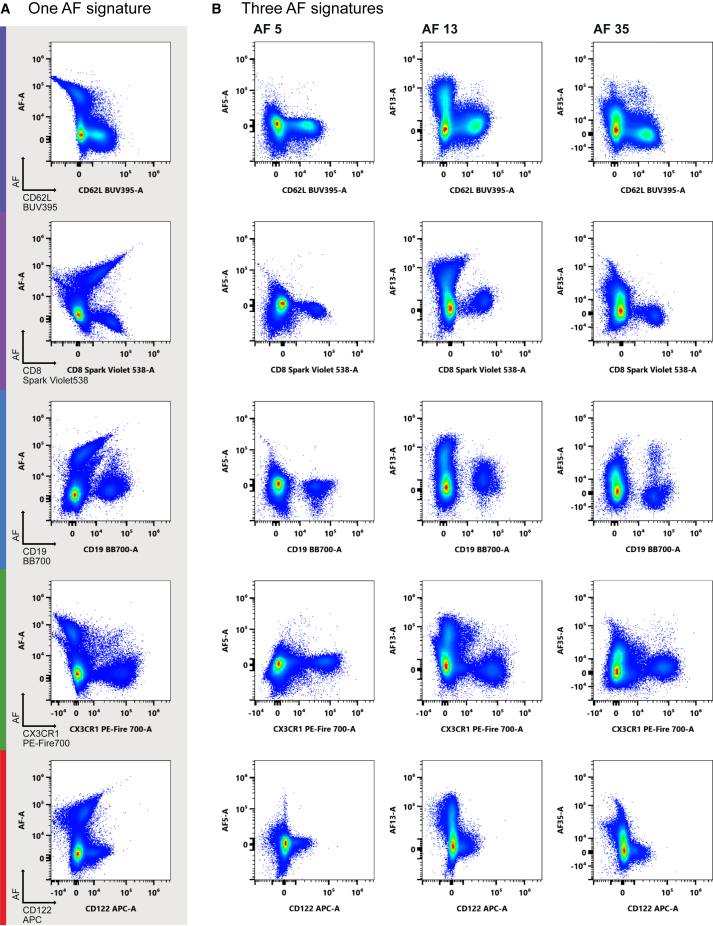
Single vs. multiple AF signatures for data unmixing (A) Dot plots of a fully stained sample unmixed with a single AF signature of the whole lung sample. (B) Dot plots from the same fully stained sample as in (A); only unmixed using the three AF signatures 5, 13, and 35. Plots display the single AF signatures vs. the indicated fluorochromes. Images show one fluorochrome example per laser, labeled with the corresponding colors.

To improve data analysis, we applied the Cytek AF dissection method by dividing an unstained lung sample into 35 AF signatures based on different forward scatter area (FSC-A) and side scatter area (SSC-A) values, as recently published.^45^ We imported all signatures separately as AF1-35 into SpectroFlo as fluorochromes and compared their similarity indices (NxN comparison). If two AF signatures were very similar, then we excluded the one with the lower spectral intensity. This allowed for the selection of 12 unique AF signatures that we added as fluorochromes to the panel of 27 colors. Next, we assessed the unmixing accuracy of all 12 AF signatures and excluded those that either did not contribute to unmixing accuracy or created inaccuracies. Not surprisingly, we found that AMØs isolated from bronchoalveolar lavage were autofluorescent in all 64 channels and interfered with our antibody panel (Figure 2A, bottom). Ultimately, we found that AF5, -13, and -35 defined the most accurate autofluorescent signals (Figure S2). AF13 was spectrally identical to the AF spectrum of AMØ, with a similarity index of 1 (Figures 2B and 2C), and was also detected in cDC2s (Figure S2B). AF5 and -35 were present across several cell subsets. Using three AF signatures for unmixing instead of one limited background signal and spread across several fluorochromes (Figure 3). This is visualized by plotting the AF signature on the y axis and comparing the data unmixing to any chosen fluorochrome on the x axis. Importantly, the improved data unmixing was independent of fluorochrome emission wavelength. Thus, the use of multiple AF signatures has helped optimize a high-parameter lung immunophenotyping panel.

### Manual cell subset analysis

**Figure 4:**
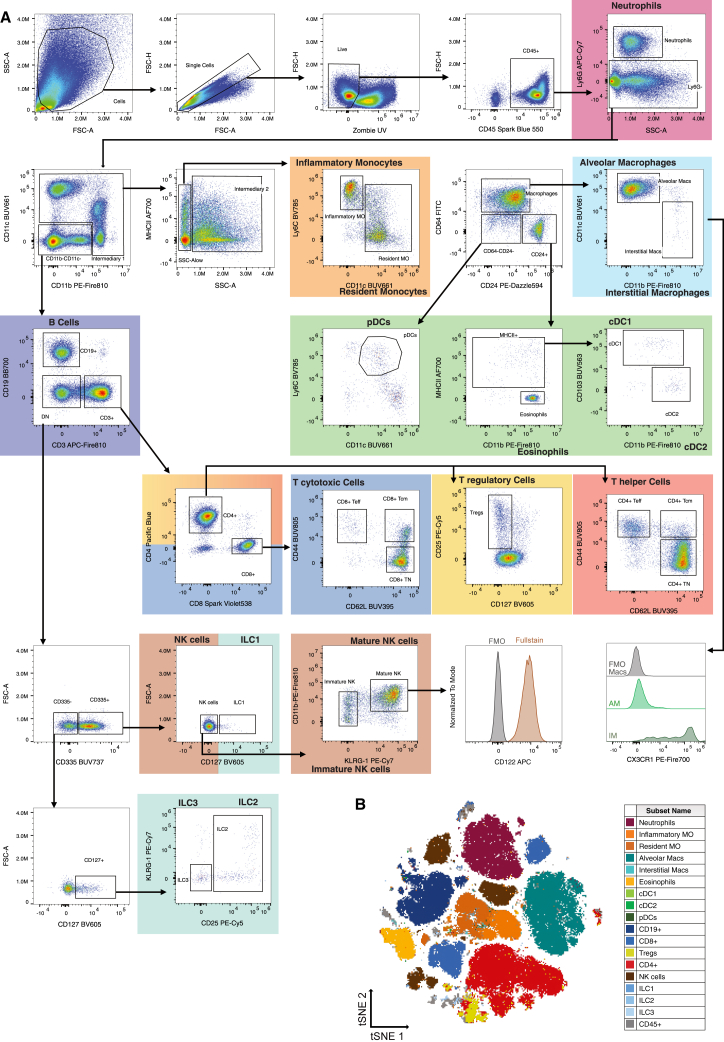
Gating strategy for identifying major immune cell-subsets Overview of the manual gating strategy from healthy lung immune cells. (A) Cells were gated to identify single, live (Zombie^−^), and CD45^+^ leukocytes. Neutrophils classified as Ly6G^+^ are underlined in purple. Ly6G^−^ cells were further plotted against CD11b and CD11c (intermediary 1) to identify myeloid cell populations. Using MHC class II^+/−^ and SSC-A^low^ cells, monocytes were identified and further subclassified to inflammatory (Ly6C^+^) and resident (CD11c^+^) monocytes, both underlined in orange. We further separated CD64^+^ macrophages from intermediary 2. The image distinguishes AMØs (CD11c^+^) from IMØs (CD11c^−^CD11b^+^), underlined in light blue. AMØs can, moreover, be distinguished from IMØs by the expression of CD206. Within the CD24^+^ gate (CD64^−^CD24^+^), we could depict eosinophils (CD11b^+^MHC class II^−^) and dendritic cells (DCs), underlined in light green. DCs are further divided into conventional DC1s (CD103^+^CD11b^−^) and cDC2s (CD11b^+^CD103^−^). Plasmacytoid DCs are identified within the CD64^−^CD24^−^ gate as CD11c^+^Ly6C^+^ cells. From the CD11b^−^CD11c^−^ gate, we identified CD19^+^ B cells (underlined in dark blue) and CD3^+^ T cells. T cells were further divided into CD8^+^ cytotoxic T cells, CD4^+^ T helper cells, and CD4^+^CD25^+^CD127^low/−^ T regulatory cells. From the CD3^−^CD19^−^ double-negative (DN) gate, CD335^+^ cells were distinguished using CD127^+^ as ILC1s (underlined in turquoise) or CD127^−^ as NK cells (underlined in brown). Mature NKs are identified as CD11b^+^KLRG-1^+^CD122^+^ cells. The CD335^−^ population was refined for CD127 expression (CD127^+^ gate) and divided into ILC2s and ILC3s by plotting CD25 vs. KLRG-1 (underlined in turquoise). Histograms are normalized to the mode due to the low abundance of IMØ. (B) tSNE analysis of a 27-color-stained sample run on single/live/CD45^+^ cells using FlowJo (BD) and overlaid with manually gated populations from (A) with the corresponding colors. The data were visually aligned for the median of the negative and positive populations of only 18 of the 870 NxN combinations where spillover was observed. AMØ, alveolar macrophages; cDC, conventional dendritic cells; FMO, fluorochrome minus one; ILC, innate lymphoid cells; IMØ, interstitial macrophages; MO, monocytes; tSNE, t-distributed stochastic neighbor embedding.

This 27-color (30-parameter) panel focuses on the main subsets of innate and adaptive immunity. For initial sample pre-processing, we excluded debris (FSC-A vs. SSC-A) and gated on single cells (FSC-H vs. FSC-A), live cells (Zombie UV^−^), and leukocytes (CD45^+^) (Figure 4A). We then identified mature (Ly6G^+/high^) neutrophils.^46^

To distinguish different myeloid cell types within the leukocyte population, we gated on CD11c^+^ or CD11b^+^ leukocytes (“intermediary 1,” Figure 4A), with further gating on SSC-A^low^MHC class II^−/+^ monocytes that can be separated further into inflammatory (Ly6C^+^) and resident (CD11c^+^) monocytes.^9^

In gate “intermediary 2” (Figure 4A), macrophages, DCs, and eosinophils could be discriminated using CD64 and CD24. Macrophages (CD64^+^) were further subdivided into AMØs (CD11c^+^) and IMØs (CD11b^+^).^9^^,^^10^^,^^47^ The two subtypes can also be segregated by the expression of CD206 in AMØs^10^ and CX3CR1 in IMØs (Figure 4A).^9^ Within the CD24^+^ gate, we identified DCs (MHC class II^+^CD11b^+^/^−^) and eosinophils (MHC class II^−^CD11b^+^). DC subclassification into cDC1s and cDC2s was based on the expression of CD11b and CD103.^11^ pDCs are classified as CD24^−^CD64^−^Ly6C^+^CD11c^+^.^10^

B cells (CD3^−^CD19^+^) and T cells (CD3^+^CD19^−^) were distinguished within the lymphocyte compartment (CD11c^−^CD11b^−/low^) (Figure 4A). T cells were further divided into CD4^+^ T helper cells and CD8^+^ cytotoxic T cells. In addition, T effector cells (T_EFFs_) were found within both T cell compartments (CD4^+^ and CD8^+^) as CD44^hi^CD62L^−^, central memory T cells (T_CMs_) as CD44^hi^CD62L^+^, and naive T cells (T_Ns_) as CD44^low^CD62L^+^.^48^ CD4^+^ T helper cells were further subclassified into T_regs_ as CD25^+^CD127^low^.

NK cells were identified as CD335^+^CD127^−^ cells and ILC1s as CD335^+^CD127^+^ (Figure 4A).^49^ Mature NK cells are CD11b^+^KLRG-1^+^CD122^+^, whereas immature NKs are CD11b^−/low^KLRG-1^−^. Among the CD335^−^CD127^+^ cells, CD25^+^ marked the ILC2 subpopulation and CD25^−^KLRG-1^−^ marked ILC3s.^49^^,^^50^

A t-distributed stochastic neighbor embedding (tSNE) dimensionality reduction was performed to cluster the data in an unbiased way (Figure 4B). Subsequently, the data were overlaid with manually gated immune cell populations to confirm the gating accuracy of the main immune cell populations.

### Panel application in mammary tumor metastatic lung samples

**Figure 5:**
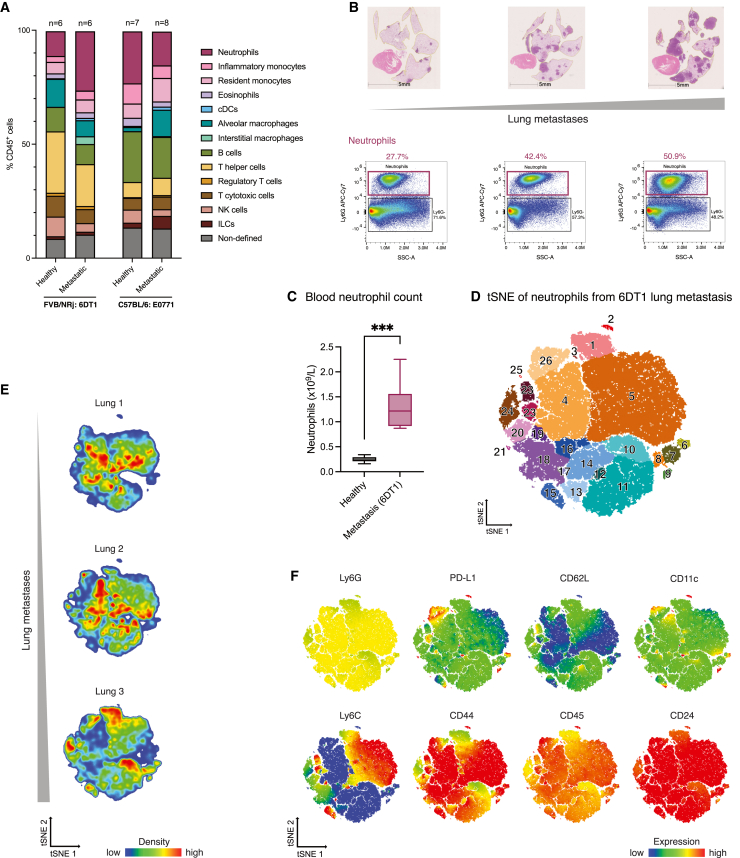
The lung immune system changes with metastases (A) Percentage of immune cell subtypes in healthy and metastasis-bearing mice identified by manual gating using the markers from Table S1. *N* denotes number of biological replicates. (B) Percentage of neutrophils per CD45^+^ cells varying over different metastatic burdens visualized by representative lung hematoxylin and eosin (H&E) staining (with the heart in pink and metastases in dark purple). Percentages are frequencies of the parent gate. (C) Hematological analysis of blood neutrophil count in healthy or metastasis-bearing FVB/NRj female mice (*n* = 4–8 per condition). Statistical test indicated by asterisk: Mann-Whitney U-test, ∗∗∗*p* < 0.001, mean ± SD. (D) Neutrophil tSNE cluster identification. Corresponding marker enrichment modeling list can be found in Table S3. (E) tSNE analysis of FVB/NRj mice bearing 6DT1 lung metastases with different numbers of lung metastases. The tSNE was run on single cells/live cells/CD45^+^/Ly6G^+^ neutrophils. Samples are arranged according to increasing metastatic nodules, assessed visually before organ processing. (F) Concatenated tSNE file of all lung samples from (D), showing different surface marker analysis on subsets. cDC, conventional dendritic cells; ILC, innate lymphoid cells; tSNE, t-distributed stochastic neighbor embedding.

Here, we used lungs with or without mammary cancer metastases to assess the applicability of our 27-antibody panel. We found that the healthy lung immune microenvironment varies depending on the mouse strain. FVB/NRj female mice have a higher percentage of CD4^+^ T helper cells, while C57BL/6 females have comparatively more neutrophils and B cells (Figure 5A). To investigate lung metastases, we intravenously injected two triple-negative metastatic mammary cancer cell lines, 6DT1 and E0771, in FVB/NRj and C57BL/6 female mice, respectively. Similar to human breast cancer heterogeneity, the two models showed divergent immune alterations (Figure 5A). 6DT1-evoked metastases associated with a higher percentage of neutrophils in the lung compared to E0771, which increased with more metastases (Figure 5B). Blood neutrophil abundance in 6DT1 lung metastases-bearing mice were significantly higher compared to healthy animals (Figure 5C).

To investigate neutrophil heterogeneity in the 6DT1 model, we performed a tSNE analysis on all Ly6G^+^ cells isolated from lungs with metastases and identified 26 clusters (Figure 5D). Mice displayed high heterogeneity in those clusters, depending on the number of lung metastases (Figure 5E). Clusters 1, 10, and 24 were composed of neutrophils with lower expression of Ly6G, CD45, and CD24 (Figure 5F), which indicated more immature neutrophils and correlated with increased lung metastases (Figure 5F; Table S3). Clusters 12 and 23 highly expressed the homing receptor CD62L. These findings reveal a diversity of neutrophils in metastatic lungs that warrants further investigation.

**Figure 6:**
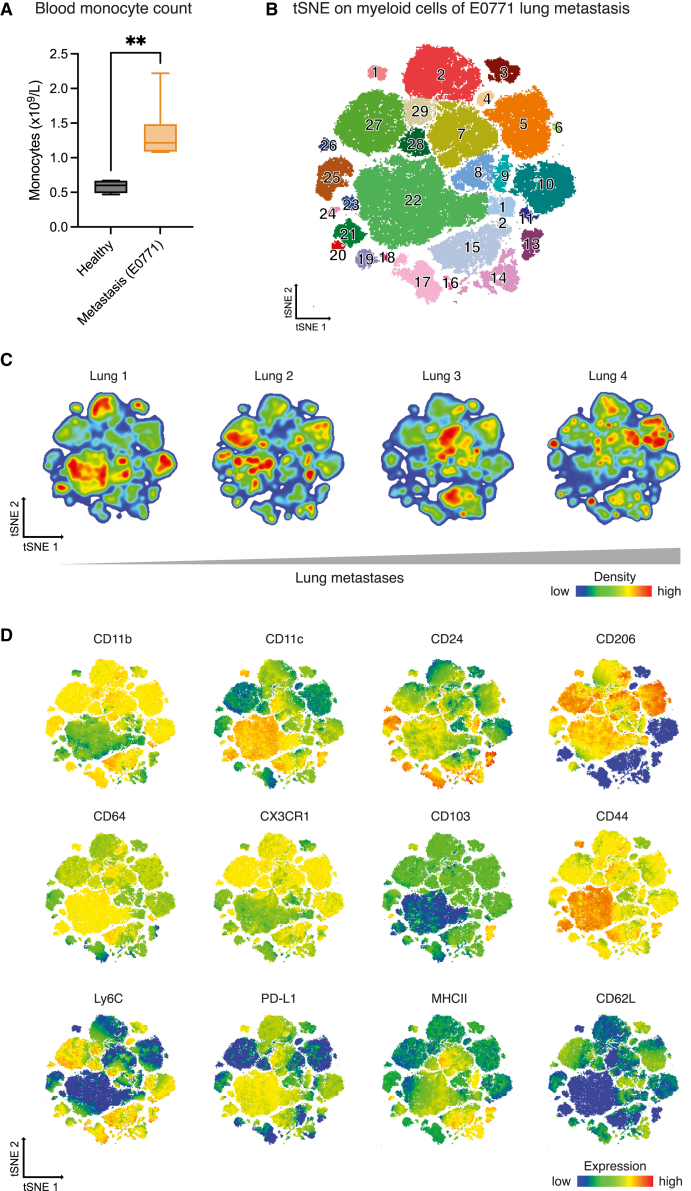
Myeloid plasticity in E0771 lung metastasis (A) Blood monocyte counts in healthy and metastases-bearing C57BL/6 females (*n* = 7–8 per condition). Statistical test indicated by asterisk: Mann-Whitney U-test, ∗∗*p* < 0.01, mean ± SD. (B) Myeloid cell tSNE myeloid (non-neutrophil) cells from E0771 metastases-bearing lungs. Data were pre-gated on cells/single cells/live cells/CD45^+^/Ly6G^−^/intermediary 1 before clustering. Population identification is displayed by the concatenated file of the metastatic lung samples. Corresponding marker enrichment modeling list can be found in Table S4. (C) tSNE from (B) from different E0771 metastases-bearing lungs. Samples are arranged according to increasing metastatic nodules, assessed visually before organ processing. (D) Concatenated tSNE file from (B) of all E0771 metastatic lung samples displaying different surface marker analysis on subsets as indicated. tSNE, t-distributed stochastic neighbor embedding.

Next, we investigated the immune diversity in the E0771 model. Metastases-bearing animals had more monocytes and macrophages in the lungs (Figure 5A) and more monocytes in the blood than healthy animals (Figure 6A).

A tSNE analysis of all lung myeloid cells but excluding neutrophils showed heterogeneity in cluster densities depending on metastatic burden (Figures 6B and 6C). For example, AMØs (cluster 22; CD64^+^CD11c^+^CD206^+^) diminished with increased metastases (Figure 6C; Table S4). Concomitantly CD11b^+^CX3CR1^+^ (e.g., clusters 5, 7, and 15) monocyte-derived macrophage populations, and MHC class II^+^CD11b^+^Ly6C^+^ cells (cluster 15), potentially consisting of tumor-associated macrophages, increased (Figures 6C and 6D). These results uncover a variety of non-neutrophil myeloid cells in metastatic lungs, highlighting the importance for further analysis.

## Discussion

In preclinical lung research, the scarcity of high-parameter flow cytometry murine lung panels has been attributed to inherent complexities arising from elevated cellular AF.^43^ To address this gap, we present a comprehensive 27-color antibody panel specifically tailored for murine lung immunophenotyping. Our panel design integrates a variety of existing strategies to create an optimal initial configuration.^51^ Despite this, we encountered challenges related to spectral interference necessitating significant optimization. Persistent background signals in the UV and violet channels were identified after classical data unmixing, prompting the strategic rearrangement of several fluorochromes across the 64 detector channels. This adjustment effectively mitigated interference from both AF and other fluorochromes.

Our panel contains multiple myeloid immune cell subtype lineage markers to unequivocally separate macrophages from monocytes, eosinophils, and DCs. However, depending on the research interest, myeloid cell markers such as CD64 could be exchanged with Siglec F and MerTK, especially for lung fibrosis or macrophage phagocytosis studies.^52^^,^^53^ Also, for a full characterization of NK cell maturation stages, CD27 may be included and plotted against CD11b.^54^

After the antibody-fluorochrome optimization, we incorporated three AF signatures using a segmentation method developed recently and employed in highly autofluorescent murine skeletal muscle samples.^45^ Notably, a semi-automated version of the AF dissection workflow implemented in SpectroFlo (from v.3.2.0 onwards) by Cytek Biosciences was not available during the completion of our panel. The SSC-A and FSC-A values provide a starting point for the identification of the AF signatures in the healthy lung. Users are advised to follow this workflow to identify specific AF signatures that may be inherent to their sample treatment and digestion.

Few studies focusing on global immunophenotyping of the murine lung were reported. A 13-color backbone panel applicable across several murine organs and spectral flow cytometry instruments^7^ was reported, but it fails to identify ∼23% of CD45^+^ cells within lungs from C57BL/6 mice. In contrast, our panel only misses 8%–13% of these cells in the healthy lungs (Figure 5A). Another study provides a 20-parameter lung panel but does not include cell activation or inhibition markers, nor does it identify regulatory T cells, ILCs, or pDCs.^8^ They include markers to discriminate γδT cells that we omitted, and none of the studies provide a means to identify NK T cells. Additionally, whereas their study defines one lung AF signature per condition, we use three.

We show that lungs with 6DT1 metastases have heterogeneous neutrophil subsets. In contrast, those with E0771 metastases harbor other myeloid cell clusters. Primary mammary tumors from 6DT1 and E0771 cancer cells cluster together by transcriptomic analysis,^55^ suggesting that the lung immune phenotype can vary even between similar models. This highlights the need to use several preclinical mouse models for immune-oncology research and to immune-profile both the primary tumor and metastases. Differences in the lung immune cell subsets can be uncovered using the herein described panel.

In summary, we report an optimized full-spectrum immunophenotyping panel for murine healthy lungs and lungs bearing mammary cancer metastases. The panel provides a prevailing tool for most major innate and adaptive immune cell populations and can be useful not only in preclinical studies on cancer immunology but also in pulmonary disorders such as bacterial or viral infections, asthma, and COPD.

### Limitations of the study

In this study, we successfully applied antibody dilutions to lung samples from both healthy and lung metastases-bearing mice. Conceivably, in other lung pathologies, immune cell infiltration may require an additional titration of antibodies to ensure optimal results. Furthermore, certain treatments, such as those involving autofluorescent compounds, may alter lung AF. We did not assess the applicability of our three AF signatures in such contexts. Additionally, any modifications to the antibody panel, whether in epitope or fluorochrome, would necessitate testing prior to application.

## Resource availability

### Lead contact

Further information and requests for resources and reagents should be directed to and will be fulfilled by the lead contact, Mohamed Bentires-Alj (m.bentires-alj@unibas.ch).

### Materials availability

The 6DT1 and E0771 murine mammary cancer cell lines were a generous gift from Dr. Lalage Wakefield. This paper did not generate any new unique reagents.

### Data and code availability

• Raw flow cytometry data files of a healthy lung (data used for Figures 2, 3, and 4) are publicly available on Mendeley Data (https://doi.org/10.17632/s5v8t53bsw.1).
• This paper does not report original code.
• Any additional information required to reanalyze the data reported in this work paper is available from the lead contact upon request.

## Acknowledgments

The authors thank Laura Ducimetière from Cytek Biosciences for help with AF segmentation and troubleshooting and members of the Bentires-Alj lab, especially Marie-May Coissieux, Markus Ackerknecht, Sok Lin Foo, and Valentina Mele, for technical advice. We are also grateful for the support given by Patrick Kury and Julia Dickow from BioLegend, by Ioannis Panetas from BD Biosciences, by Baptiste Hamelin for critically reading the manuscript, and by past and present DBM Flow Cytometry Core Facility members for machine maintenance and continuous support. Our gratitude goes to the DBM Histology Core Facility, especially Diego Calabrese and Mylène Toranelli for support and performing the H&E staining. Also, we are highly appreciative of the whole DBM animal core facility team. In addition, we thank Martina Konantz for her help with the hematology blood analysis. Figures were partially created with BioRender.com. Z.B. was supported by the Dr. Arnold U. and Susanne Huggenberger-Bischoff Foundation for Cancer Research, the Peter Bockhoff Foundation, the Fondation Bryn Turner-Samuels, the Pedersen Charity Foundation, the Hedy-Glor-Meyer Foundation, the 10.13039/100009736Freiwillige Akademische Gesellschaft, Basel, and the 10.13039/501100022796Jubiläumsstiftung von Swiss Life. M.B.-A. was supported by the 10.13039/501100000781European Research Council (ERC advanced grant 694033 STEM-BCPC), the 10.13039/501100001711Swiss National Science Foundation, the 10.13039/501100006069Krebsliga Beider Basel, the 10.13039/501100004361Swiss Cancer League (KFS-4414-02-2018), the Swiss Personalized Health Network (Swiss Personalized Oncology driver project), and the Department of Surgery of the University Hospital Basel.

## Author contributions

Conceptualization, Z.B., C.W., T.L., and M.B.-A.; methodology, Z.B.; validation, Z.B. and C.M.V.; formal analysis, Z.B., C.M.V., and V.R.; investigation, Z.B. and C.M.V.; resources, M.B.-A.; data curation, Z.B.; writing – original draft, Z.B.; writing – review & editing, Z.B., C.W., C.M.V., T.L., and M.B.-A.; visualization, Z.B.; supervision, T.L. and M.B.-A.; funding acquisition, Z.B. and M.B.-A.

## Declaration of interests

C.W. is a current employee of BioLegend, Inc.

## STAR★Methods

### Key resources table

**Table:** 

REAGENT or RESOURCE	SOURCE	IDENTIFIER
**Antibodies**		

Anti-Mouse CD16/CD32 (FcBlock); Clone: 2.4G2; 500 ng/test	BD Biosciences	Cat#553142; RRID: AB_394656
Zombie UV Fixable Viability Dye; 1:400	BioLegend	Cat#423108; RRID: N/A
CD62L – BUV395; Clone: MEL-14; 50 ng/test	BD Biosciences	Cat#740218; RRID: AB_2739966
CD103 – BUV563; Clone: M290; 100 ng/test	BD Biosciences	Cat#741261; RRID: AB_2870808
CD11c – BUV661; Clone: N418; 50 ng/test	BD Biosciences	Cat#750449; RRID: AB_2874610
CD335 (Nkp46) – BUV737; Clone: 29A1.4; 100 ng/test	BD Biosciences	Cat#612805; RRID: AB_2870131
CD44 – BUV805; Clone: IM7; 33 ng/test	BD Biosciences	Cat#741921; RRID: AB_2871234
F4/80 – BV421; Clone: BM8; 200 ng/test	BioLegend	Cat#123132; RRID: AB_11203717
CD4 – Pacific Blue; Clone: GK1.5; 83 ng/test	BioLegend	Cat#100428; RRID: AB_493647
CD8 – Spark Violet538; Clone: QA17A07; 250 ng/test	BioLegend	Cat#155020; RRID: AB_2890706
CD127 (IL7Rα)– BV605; Clone: A7R34; 200 ng/test	BioLegend	Cat#135025; RRID: AB_2562114
CD206 (MMR) – BV650; Clone: C068C2; 100 ng/test	BioLegend	Cat#141723; RRID: AB_2562445
CD274 (PD-L1) – BV711; Clone: MIH5; 14 ng/test	BD Biosciences	Cat#563369; RRID: AB_2738163
Ly6C – BV785; Clone: HK1.4; 25 ng/test	BioLegend	Cat#128041; RRID: AB_2565852
CD64 – FITC; Clone: X54-5/7.1; 500 ng/test	BioLegend	Cat#139316; RRID: AB_2566556
CD45 – Spark Blue550; Clone: 30-F11; 100 ng/test	BioLegend	Cat#103166; RRID: AB_2832300
CD19 – BB700; Clone: 1D3, 25 ng/test	BD Biosciences	Cat#566411; RRID: AB_2744315
CD152 (CTLA-4) – PE; Clone: UC10-4B9; 100 ng/test	BioLegend	Cat#106306; RRID: AB_313255
CD24 – PE-Dazzle594; Clone: M1/69; 50 ng/test	BioLegend	Cat#101838; RRID: AB_2566732
CD25 – PE-Cy5; Clone: PC61; 100 ng/test	BioLegend	Cat#102010; RRID: AB_312859
CX3CR1 – PE-Fire700; Clone: SA011F11; 25 ng/test	BioLegend	Cat#149052; RRID: AB_2910299
KLRG-1 – PE-Cy7; Clone: 2F1/KLRG1; 50 ng/test	BioLegend	Cat# 138416; RRID: AB_2561736
CD11b – PE-Fire810, Clone: M1/70; 33 ng/test	BioLegend	Cat# 101285; RRID: AB_2904271
CD122 (IL-2Rβ) – APC, Clone: TM-β1; 100 ng/test	BioLegend	Cat#123214; RRID: AB_2562575
CD279 (PD-1) – AF647; Clone: 29F.1A12; 250 ng/test	BioLegend	Cat# 135230; RRID: AB_2566008
I-A/I-E (MHC class II) – AF700; Clone: M5/114.15.2; 63 ng/test	BioLegend	Cat# 107622; RRID: AB_493727
Ly6G – APC-Cy7; Clone: 1A8; 20 ng/test	BioLegend	Cat#127624; RRID: AB_10640819
CD3 – APC-Fire810; Clone: 17A2; 100 ng/test	BioLegend	Cat#100268; RRID: AB_2876392
CD45 – AF700; Clone: X54-5/7.1	BioLegend	Cat#103128 RRID: AB_493715
CD64 – FITC: 30-F11	BioLegend	Cat#139316 RRID: AB_2566556
CD11c – PerCP-Cy5.5; Clone: N418	BioLegend	Cat#117327 RRID: AB_2129641

**Biological samples**		

Mouse lung immune cells	Internal breeding	N/A
Mouse whole blood	Internal breeding	N/A

**Chemicals, peptides, and recombinant proteins**		

Phosphate Buffered Saline, pH 7.4 (PBS)	Gibco, Thermo Fisher	Cat#10010023
Fetal Bovine Serum (FBS) double heat-inactivated at 56°C for 30 min	Sigma Aldrich	Cat#F7524
RPMI-1640 Medium	Sigma Aldrich	Cat#R8758-500ML
Dulbecco’s Modified Eagle’s Medium (DMEM)	Sigma Aldrich	Cat#D6429-500ML
HEPES buffer solution	Sigma Aldrich	Cat#83264-100ML-F
Ethylenediaminetetraacetic 0.5 M Solution (EDTA)	Gerbu	Cat#1534
Dimethyl Sulphoxide Hybri-Max (DMSO)	Merck	Cat#D2650-100ML
Paraformaldehyde 32% (PFA)	Electron Microscopy Science, Thermo Fisher	Cat#50-980-495
Formal-Fixx	Epredia	Cat#9990244
Trypan Blue Stain 0.4%	Gibco, Thermo Fisher	Cat#15250061
DNAse I (Type IV; 2.5 KU/mL); 10 μL/mL	Sigma Aldrich	Cat#D5025-15KU
Liberase DL Research Grade; 5 mg/mL	Roche, Merck	Cat#5401160001
Red Blood Cell Lysis Buffer	Roche Diagnostics, Merck	Cat#11814389001
BD Horizon Brilliant Stain Buffer	BD Biosciences	Cat#566349
UltraComp eBeads Plus	Life Technologies, Thermo Fisher	Cat#01-3333-42
SpectroFlo QC beads 2000 Series	Cytek Biosciences	Cat#N7-97355

**Experimental models: Cell lines**		

6DT1	Gifted by Dr. Lalage Wakefield	N/A
E0771	Gifted by Dr. Lalage Wakefield	N/A

**Experimental models: Organisms/strains**		

Mus musculus, FVB/NRj	In house breeding	N/A
Mus musculus, C57Bl/6NCrl	In house breeding	N/A

**Software and algorithms**		

SpectroFlo	Cytek Biosciences	N/A
FlowJo 10.10.0	BD Biosciences	N/A
tSNE FlowJo Plugin 2.2	BD Biosciences	N/A
Affinity Designer 2.4.2	Serif	N/A
HALO	Indica Labs	N/A
Prism 10.2.3	GraphPad	N/A
R 4.4.0	R Development Core Team	N/A

**Other**		

Cytek Aurora 5-Laser-V16-B14-YG10-UV16	Cytek Biosciences	N/A
Sysmex XE-5000 analyzer	Sysmex	N/A

### Experimental model and study participant details

#### Animals

All *in vivo* experiments were performed following the Swiss animal welfare ordinance and approved by the cantonal veterinary office Basel-Stadt, Switzerland. Adult (7–14 weeks) female FVB/NRj and C57BL/6 mice were housed in the Department of Biomedicine animal facility. Mice were maintained in an SPF-free environment with light, humidity, and temperature control (12-h light–dark cycle at 21°C–25°C and a humidity of 45–65%). Animals were co-housed with 3–6 animals per cage, *ad libitum* access to food and water, and a red house (Tecniplast) for enrichment.

#### Cancer cell line

The 6DT1 and E0771 murine mammary cancer cell lines were a generous gift from Dr. Lalage Wakefield.^55^ 6DT1 were cultured in DMEM (Sigma Aldrich, Cat#D6429-500ML), supplemented with 10% FBS (Sigma Aldrich; Cat#F7524), 1% Penicillin-Streptomycin (Sigma Aldrich, Cat#P4333), and 100 μg/mL Normocin (Labforce, Cat#ant-nr-1). E0771 were cultured in RPMI-1640, supplemented with 5% FBS, 1% Penicillin-Streptomycin, and 10 mM HEPES (Sigma Aldrich, Cat#83264). The cell lines were regularly tested for Mycoplasma contamination.

### Method details

#### Isolation of mouse lung immune cells

(1) Isolate lungs from a sacrificed mouse into cold RPMI medium, store on ice. Remove all parts of the thymus and other non-lung tissue.
(2) Transfer the lungs into gentleMACS C tubes containing 1 mL RPMI and cut them into fragments (2 mm or smaller) with scissors.
(3) Enzymatic digestion: Add 2 mL digestion mix (RPMI supplemented with 5 μL/mL Liberase and 10 μL/mL DNAse I) per sample and incubate at 37°C for 25 min in an orbital shaker. Stop digestion by adding 3 mL RPMI and place the samples on ice.
(4) Mechanical dissociation: Homogenize sample further in the gentleMACS C tubes using the gentleMACS Octo Dissociator (1302 rounds per run; 55 s).
(5) Mash remaining lung fragments with the plunger of a syringe over a 70-μM strainer on top of a 50-mL conical tube. Wash thoroughly with cold flow cytometry buffer (FB) to minimize cell loss and centrifuge (250 x *g* for 5 min at 4°C); aspirate supernatant.
(6) Lyse pellet with 1 mL red blood cell lysis buffer, vortex, and incubate 1–2 min at room temperature (RT).
(7) Stop reaction by adding 10 mL FB, centrifuge (250 x *g* for 5 min at 4°C) and aspirate supernatant.
(8) Resuspend pellet in 1 mL FB.
(9) Count the total number of live cells in suspension with a hemocytometer and trypan blue and transfer 2 million live cells into a 96-well plate. The leftover cells are used for FMO staining.
(10) Centrifuge at 250 x *g* for 5 min at 4°C and invert plate.
(11) Wash samples twice with 200 μL PBS and spin down at 250 x *g* for 5 min at 4°C.

#### Viability staining

(1) Thaw an aliquot of Zombie UV (lyophilized dye is resuspended in DMSO as per manufacturer’s recommendation and 5 μL aliquots are stored at −20°C protected from light).
(2) Prepare Zombie UV dilution in PBS in amber tubes according to dilution needed (in our case 1:400).
(3) Resuspend cells in 50 μL Zombie UV mixture (as prepared in step 2) and incubate for 10 min at RT in the dark. Resuspend unstained cells in 50 μL PBS.
(4) Add 150 μL PBS to all wells and centrifuge the plate (250 x *g*, 5 min, 4°C); remove supernatant by flicking the plate upside down.
(5) Resuspend samples in 50 μL FcBlock in FB (1:100) and incubate 10 min at 4°C in the dark.
(6) Continue immediately with surface staining (paragraph below).

#### Surface staining

(1) Spin antibodies for 10 s in a microcentrifuge at 1200 x *g* to spin down aggregates.
(2) Prepare full panel and fluorochrome minus one (FMO) mixes on ice in amber 1.5 mL tubes to avoid light exposure. Pipet pre-titrated antibodies into brilliant stain buffer to a total volume of 50 μL per sample and FMO. Keep on ice.
(3) Add 50 μL of the corresponding antibody master mix per sample (on top of the prelaid 50-μL FcBlock mixture); pipet up and down.
(4) Incubate for 30 min on ice protected from light.
(5) Wash cells twice with 200 μL FB, pellet by centrifugation (250 x *g*, 5 min, 4°C), and invert plate to remove supernatant. Of note, brilliant stain buffer is used to alleviate non-specific reactivity of polymers, possibly resulting in false-positive staining artifacts.^56^

#### Sample fixation

Samples were fixed to allow flexibility of data acquisition.

(1) Fix cells by resuspension in 200 μL of 2% PFA and incubate them for 20 min at RT in the dark.
(2) Pellet samples by centrifuging at 600 x *g* for 5 min at 4°C and invert plate to remove supernatant.
(3) Resuspend cell pellets in 200 μL FB and store on ice protected from light until acquisition.
(4) Analyze samples on a Cytek Aurora.

It is anticipated that intracellular staining is easily implementable with minimal adjustments.

#### Single stain controls

Unmixing was performed using beads stained with the same antibody-fluorochrome conjugates as the full-stained and FMO samples. Fluorochromes can show different spectral characteristics when coupled to beads instead of cells,^15^ beads were assessed initially for the following reasons: Due to the many required FMOs in this panel, there were not enough leftover cells for unmixing using isolated lung cells. Following the 3R guidelines, we support the minimal use of lab animals.^57^ Moreover, the use of beads guaranteed a high positive signal for proper selection of positive events. Nevertheless, we tested unmixing using single stained cells. For most antibody-fluorochrome conjugates, unmixing fluorochromes in the ultraviolet and violet channels such as BV711 led to unmixing issues (data not shown). However, for AF700, APC and PE-Fire810, the use of cells instead of beads alleviated some unmixing errors and we continued to use cells for unmixing with these three markers. Finally, there were no critical unmixing errors that could not be visually aligned in the final panel setup and all populations were identified by manual gating or by unsupervised clustering (Figure 4).

#### Staining protocol of single stained controls

For single stains, five drops of UltraComp eBeads Plus were diluted in 1′500 μL PBS and distributed as 50-μL aliquots in a 96-well plate. Then 1 μL of the corresponding fluorochrome-conjugated antibody was added and incubated for 20 min on ice in the dark. After adding 150 μL FB, the beads were centrifuged at 600 x *g* for 5 min at 4°C and the supernatant removed. Fixation of beads was performed with 200 μL fixation solution (as for cellular samples) for 20 min at RT in the dark. Beads were then centrifuged at 600 x *g* for 5 min at 4°C, inverted, and resuspended in 200 μL FB for acquisition. All bead processing was performed under the same conditions as the cellular samples, e.g., temperature, light exposure, time, and fixation.^58^

#### Isolation of AMØ

Mice were euthanized using a pentobarbital (Esconarkon; 250 mg/kg diluted in 0.9% NaCl) overdose. The extra-thoracic part of the trachea was exposed, and a catheter (Insyte 22GA, BD #381423) was placed in the trachea for subsequent bronchoalveolar lavage. Briefly, the lung was washed 8–12 times with 0.8–1 mL of 4°C FB buffer whilst gently massaging the thorax. The aspirated bronchoalveolar lavage fluid (BALF) was collected and stored on ice until further processing. Samples were centrifuged at 300 x *g* for 10 min and the supernatant collected. The pellet was resuspended in 1 mL red blood cell lysis buffer (Roche Diagnostics, Merck; Cat. #11814389001) and incubated for 1–2 min at RT. Incubation was stopped by adding 4 mL of FB and samples centrifuged again at 300 x *g* for 7 min. Sample purity for AM∅ was assessed by staining the BALF with anti-mouse CD45-AF700 (BioLegend, Cat#103128), anti-mouse CD64-FITC (BioLegend, Cat#139316), and anti-mouse CD11c-PerCP-Cy5.5 (Biolegend, Cat#117327) for 30 min at 4°C and washing twice with FB and acquiring the sample on a Cytek Aurora.

#### Generation of mammary lung metastases

Adult (8–9 weeks old) female FVB/NRj and C57BL/6 mice were injected with 5x10^4^ 6DT1 or 3x10^5^ E0771 syngeneic cancer cells in 100 μL PBS in the lateral tail vein.^55^ Endpoints were reached 24 or 31 days after injection respectively, at which the mice were sacrificed by CO_2_ inhalation and the lungs were harvested and processed as described.

#### Hematology analysis

Blood was collected immediately after sacrifice by cardiac puncture into EDTA-coated tubes (Microvette 500 EDTA K3E, Sarstedt #20.1341.100) and afterward diluted 1:2 with 0.9% NaCl (Fresenius Kabi #ZYA1901). Analysis was performed on an XE-5000TM hematology analyzer (Sysmex). Blood counts displayed are from independent experiments.

#### Lung histology

Isolated lungs (and heart for orientation purposes) were perfused with PBS through the trachea with a gavage needle to remove as much blood as possible. The lungs were fixed with Formal-Fixx (Epredia) for 24 hat RT, washed with water to remove formalin, and stored in 70% ethanol at 4°C until paraffin embedding (Tissue Processing Center TPC 15 Duo, Medite). The blocks were sliced into 3.5 μm sections (Microtom HM355S, Thermo Scientific) and mounted (Superfost Microscope Slides, Epredia, #AA00008432E01MNZ20). Hematoxylin and eosin (H&E) staining was performed using a Gemini AS Slide Stainer Autostainer (Epredia); the slides were scanned with a Nanozoomer S60 (Hamamatsu) and analyzed subsequently by HALO (Indica Labs).

#### Flow cytometry data analysis

Data were analyzed and visualized using FlowJo 10.10.0 (BD) with embedded plugins tSNE 2.0.0 and marker enrichment modeling (MEM). tSNE parameters were set as follows: iterations (1000), perplexity (30–50), and eta (8218). For tSNE on CD45^+^ immune cells, only lineage markers were chosen as clustering parameters. For myeloid and neutrophil tSNEs, all markers known to be expressed on the corresponding cell populations were used. Violin plots were generated using R 4.4.0 by comparing manually gated populations for their expression of the respective AF signatures. Boxplots were generated using Prism 10.2.3 (Graphpad Software).

### Quantification and statistical analysis

Statistical analyses were performed using Prism (GraphPad Software, v10.2.3). For data comparison between two conditions, the Mann-Whitney U-test was used for all statistical analyses. A *p*-value of <0.05 was considered statistically significant; non-significant (ns) *p* > 0.05; ∗*p* < 0.05; ∗∗*p* < 0.01; ∗∗∗*p* < 0.001; ∗∗∗∗*p* < 0.0001. Data are shown as mean ± standard deviation (SD). Replicates are individual mice and numbers are indicated in the figure legends.

## Supplemental information

Document S1. Figures S1 and S2 and Tables S1–S4

Document S2. Article plus supplemental information
