# Supplementary material for: Optimized full-spectrum flow cytometry panel for deep immunophenotyping of murine lungs
Source: Cell Rep Methods. 2024 Oct 30;4(11):100885. doi: 10.1016/j.crmeth.2024.100885 (PMC11705587; doi:10.1016/j.crmeth.2024.100885)
Supplement: Document S1. Figures S1 and S2 and Tables S1–S4 [file mmc1.pdf]

Cell Reports Methods, Volume *4*

Supplemental information

Optimized full-spectrum flow cytometry panel for deep immunophenotyping of murine lungs

Zora Baumann, Carsten Wiethe, Cinja M. Vecchi, Veronica Richina, Telma Lopes, and Mohamed Bentires-Alj

A

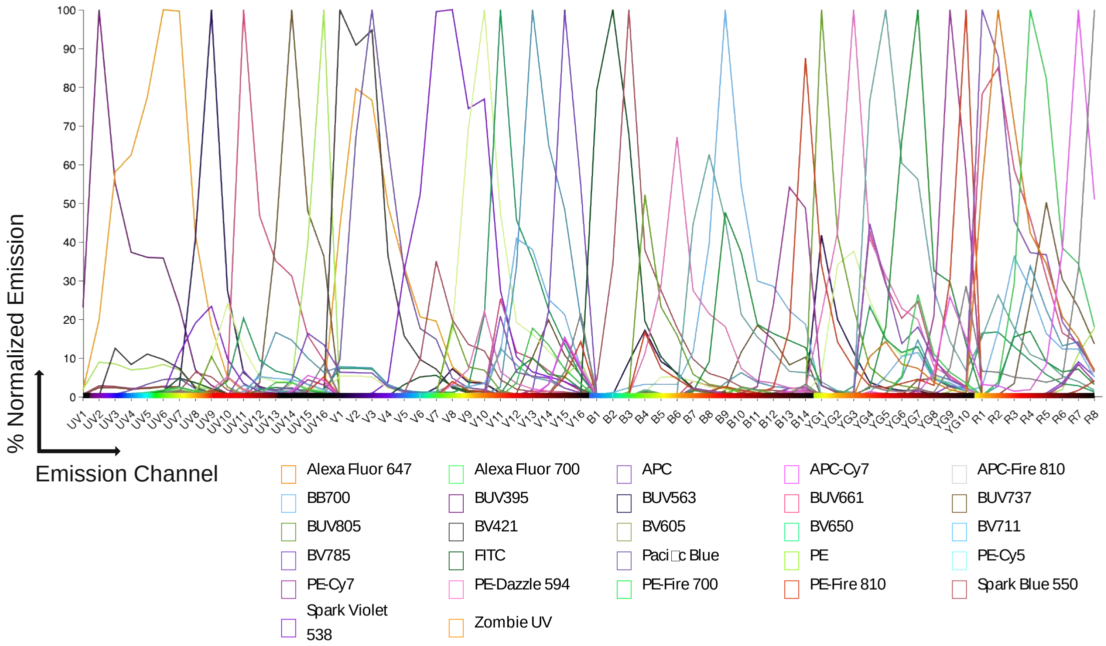

B

	BUV395	BUV563	BUV661	BUV737	BUV805	BV421	Pacific Blue	BV605	BV650	BV711	BV785	FITC	Spark Blue 550	BB700	PE	PE-Dazzle594	PE-Cy5	PE-Fire 700	PE-Cy7	APC	Alexa Fluor 647	Alexa Fluor 700	APC-Cy7	APC-Fire 810	Zombie UV	Spark Violet 538	PE-Fire 810
BUV395	1.00																										
BUV563	0.10	1.00																									
BUV661	0.05	0.06	1.00																								
BUV737	0.05	0.03	0.36	1.00																							
BUV805	0.15	0.04	0.12	0.46	1.00																						
BV421	0.06	0.01	0.00	0.01	0.02	1.00																					
Pacific Blue	0.02	0.01	0.00	0.00	0.01	0.78	1.00																				
BV605	0.01	0.19	0.19	0.06	0.02	0.09	0.08	1.00																			
BV650	0.01	0.04	0.44	0.13	0.04	0.13	0.11	0.56	1.00																		
BV711	0.01	0.01	0.28	0.39	0.14	0.16	0.13	0.19	0.47	1.00																	
BV785	0.01	0.01	0.06	0.22	0.25	0.11	0.09	0.08	0.18	0.52	1.00																
FITC	0.01	0.09	0.00	0.00	0.00	0.01	0.01	0.02	0.01	0.00	0.00	1.00															
Spark Blue 550	0.01	0.19	0.01	0.01	0.01	0.02	0.05	0.16	0.06	0.03	0.01	0.68	1.00														
BB700	0.00	0.03	0.33	0.28	0.06	0.00	0.00	0.18	0.38	0.55	0.23	0.03	0.10	1.00													
PE	0.00	0.46	0.04	0.01	0.00	0.00	0.01	0.25	0.05	0.01	0.00	0.08	0.23	0.06	1.00												
PE-Dazzle594	0.00	0.22	0.16	0.03	0.01	0.00	0.00	0.47	0.19	0.04	0.01	0.04	0.15	0.19	0.46	1.00											
PE-Cy5	0.00	0.05	0.46	0.11	0.02	0.00	0.00	0.22	0.30	0.15	0.03	0.01	0.06	0.48	0.12	0.46	1.00										
PE-Fire 700	0.00	0.04	0.27	0.18	0.04	0.00	0.00	0.14	0.18	0.25	0.07	0.01	0.04	0.56	0.08	0.28	0.64	1.00									
PE-Cy7	0.00	0.01	0.05	0.14	0.09	0.00	0.00	0.04	0.05	0.11	0.16	0.00	0.02	0.19	0.03	0.07	0.15	0.34	1.00								
APC	0.02	0.00	0.81	0.19	0.03	0.04	0.05	0.14	0.36	0.21	0.03	0.02	0.03	0.31	0.03	0.16	0.51	0.26	0.05	1.00							
Alexa Fluor 647	0.00	0.01	0.75	0.22	0.04	0.00	0.00	0.03	0.20	0.21	0.02	0.00	0.00	0.35	0.01	0.06	0.36	0.22	0.04	0.89	1.00						
Alexa Fluor 700	0.00	0.00	0.42	0.47	0.10	0.00	0.00	0.05	0.17	0.47	0.12	0.01	0.01	0.40	0.01	0.04	0.22	0.33	0.10	0.46	0.56	1.00					
APC-Cy7	0.00	0.00	0.15	0.30	0.26	0.00	0.00	0.02	0.06	0.22	0.24	0.00	0.00	0.17	0.00	0.02	0.08	0.14	0.27	0.18	0.21	0.41	1.00				
APC-Fire 810	0.00	0.01	0.14	0.22	0.31	0.00	0.00	0.03	0.07	0.17	0.24	0.00	0.01	0.14	0.02	0.03	0.09	0.12	0.23	0.17	0.18	0.29	0.74	1.00			
Zombie UV	0.46	0.38	0.08	0.07	0.14	0.36	0.34	0.08	0.07	0.07	0.05	0.03	0.05	0.01	0.02	0.01	0.00	0.00	0.00	0.05	0.00	0.00	0.00	0.01	1.00		
Spark Violet 538	0.02	0.15	0.04	0.02	0.01	0.07	0.17	0.56	0.23	0.08	0.04	0.05	0.34	0.08	0.14	0.09	0.02	0.01	0.01	0.03	0.00	0.01	0.00	0.01	0.16	1.00	
PE-Fire 810	0.00	0.10	0.05	0.10	0.09	0.00	0.00	0.08	0.05	0.08	0.11	0.02	0.06	0.17	0.22	0.15	0.15	0.29	0.73	0.05	0.03	0.07	0.17	0.26	0.01	0.03	1.00

Scale: 0 – 1

Complexity Index: 9.46

Supplementary Figure 1: Similarity indices and spectral overview (related to Figure 1)

(a) Spectral view of the 27-color panel calculated by the Cytek Full Spectrum Viewer (https://spectrum.cytekbio.com/) for the Cytec Aurora 5-laser configuration (16UV-16V-14B-10YG-8R) for the panel configuration. (b) Similarity and complexity indices from the panel configuration. Similarity Index (SI) measures how similar two given fluorochromes are to predict their spillover. From a range of 0 to 1, where 0

denotes no spillover and 1 very probable spillover due to high spectral similarity. Complexity index is the sum of the SI of a given panel. **(a)** Similarity and complexity indices generated from data generated with the used antibodies specified in the Key Resource Table and Table 2. Color coding applied as follows: white signifies no interaction and the darker the blue, the higher the interaction.

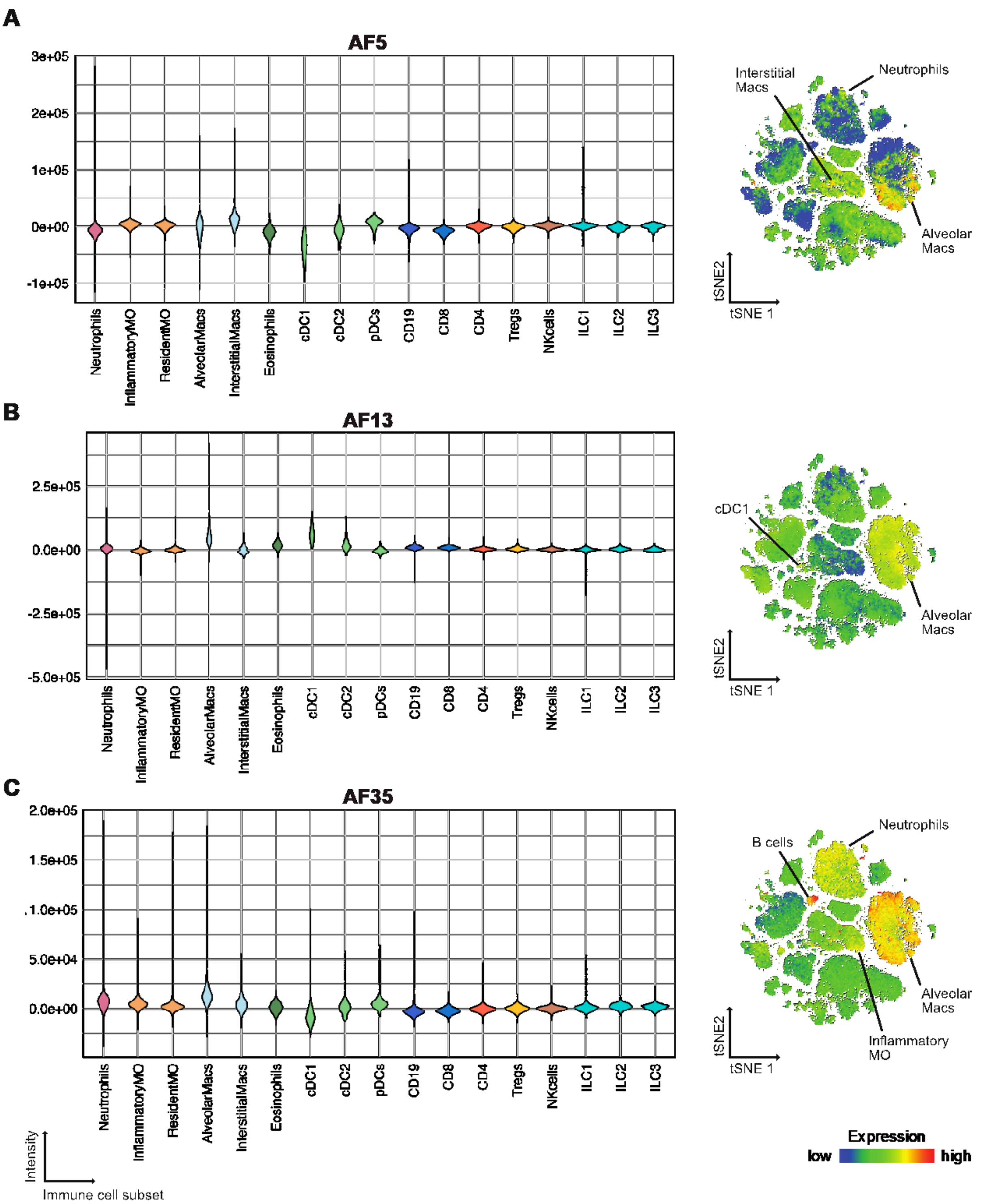

Supplementary Figure 2: Distribution of autofluorescent signatures (related to Figure 2)

(a-c) Violin plots in the left column show the intensity of AF signatures 5, 13, and 35 per immune cell subset. Right column shows tSNE for all $CD45^+$ immune cells, as displayed in Figure 4b. Color gradient shown for the corresponding AF signatures. The immune cell populations with the highest corresponding AF signatures are manually annotated. AF, autofluorescence; MO, monocytes; Macs, macrophages; cDC, conventional dendritic cells; tSNE, t-distributed Stochastic Neighbor Embedding.

Cell type	Lineage antigen markers
Neutrophils	$Ly6G^{+}$
Monocytes	$Ly6G^{-}$ $SSC\text{-}A^{low}$ $MHCII^{-/int}$
Inflammatory monocytes	$Ly6G^{-}$ $SSC\text{-}A^{low}$ $MHCII^{-/int}$ $Ly6C^{+}$
Resident monocytes	$Ly6G^{-}$ $SSC\text{-}A^{low}$ $MHCII^{-/int}$ $Ly6C^{+/-}$ $CD11c^{+}$
Eosinophils	$Ly6G^{-}$ $CD24^{+}$ $CD11b^{+}$ $MHCII^{-}$
Dendritic cells	$Ly6G^{-}$ $CD64^{-}$
cDC1	$CD24^{+}$ $MHCII^{+}$ $CD103^{+}$
cDC2	$CD24^{+}$ $MHCII^{+}$ $CD103^{-}$ $CD11b^{+}$
pDC	$CD24^{-}$ $CD11c^{+}$ $Ly6C^{+}$
Macrophages	$Ly6G^{-}$ $CD11c^{+/-}$ $CD11b^{+/-}$ $CD64^{+}$ $CD24^{-/low}$
Alveolar macrophages	$CD11b^{-}$ $CD11c^{+}$ $CD206^{+}$ $CX3CR1^{-}$
Interstitial macrophages	$CD11b^{+}$ $CD11c^{-/int}$ $CD206^{-}$ $CX3CR1^{+}$
B cells	$Ly6G^{-}$ $CD11c^{-}$ $CD11b^{-/int}$ $CD3^{-}$ $CD19^{+}$
T cells	$Ly6G^{-}$ $CD11c^{-}$ $CD11b^{-/int}$ $CD19^{-}$ $CD3^{+}$
Cytotoxic T cells	$CD8^{+}$
T helper cells	CD4 $^{+}CD25^{-}$
Regulatory T cells	$CD4^{+}$ $CD25^{+}$ $CD127^{-/low}$
Naïve (T_N)	$CD62L^{+}$ $CD44^{-}$
Effector memory (T_{EFF})	$CD62L^{-}$ $CD44^{+}$
Central memory (T_{CM})	$CD62L^{+}$ $CD44^{+}$
NK cells	$CD11c^{-}$ $CD11b^{-/int}$ $CD19^{-}$ $CD3^{-}$ $CD335^{+}$ $CD127^{-}$
Immature	$CD11b^{-}$ $KLRG1^{-}$
Mature	$CD11b^{+}$ $KLRG1^{+}$ $CD122^{+}$
Innate lymphoid cells (ILC)	$CD11c^{-}$ $CD11b^{-/int}$ $CD19^{-}$ $CD3^{-}$ $CD127^{+}$
ILC1	$CD335^{+}$
ILC2	$CD335^{-}$ $CD25^{+}$ $KLRG\text{-}1^{+/-}$
ILC3	$CD335^{-}$ CD25- $KLRG\text{-}1^{-}$

Supplementary Table 1: **Cell identification markers (related to Figure 4)**

List of identified cell types and subsets with corresponding lineage antigen markers. For subsets, we only listed markers other than those of the lineage. Int, intermediary.

Specificity	AB Clone	Antigen Classification	Fluorochrome	Vendor	Cat. #	Titer (ng/test)
CD62L	MEL-14	2	BUV395	BD Biosciences	740218	50
Viability	-	-	Zombie UV	BioLegend	423108	1:400
CD103	M290	3	BUV563	BD Biosciences	741261	100
CD11c	N418	2	BUV661	BD Biosciences	750449	50
CD335 (Nkp46)	29A1.4	2	BUV737	BD Biosciences	612805	100
CD44	IM7	2	BUV805	BD Biosciences	741921	33
F4/80	BM8	3	BV421	BioLegend	123132	200
CD4	GK1.5	1	Pacific Blue	BioLegend	100428	83
CD8	QA17A07	1	Spark Violet 538	BioLegend	155020	250
CD127	A7R34	3	BV605	BioLegend	135025	200
CD206 (MMR)	C068C2	3	BV650	BioLegend	141723	100
CD274 (PD-L1)	MIH5	2	BV711	BD Biosciences	563369	14
Ly6C	HK1.4	2	BV785	BioLegend	128041	25
CD64	X54-5/7.1	2	FITC	BioLegend	139316	500
CD45	30-F11	1	Spark Blue 550	BioLegend	103166	100
CD19	6D5	1	BB700	BD Biosciences	566411	25
CTLA-4	UC10-4B9	3	PE	BioLegend	106306	100
CD24	M1/69	2	PE-Dazzle 594	BioLegend	101838	50
CD25	PC61	3	PE-Cy5	BioLegend	102010	100
CX3CR1	SA011F11	2	PE-Fire700	BioLegend	149052	25
KLRG-1	2F1/KLRG1	2/3	PE-Cy7	BioLegend	138416	50
CD11b	M1/70	1	PE-Fire810	BioLegend	101285	33
CD122	TM-β1	3	APC	BioLegend	123214	100
CD279 (PD-1)	29F.1A12	3	Alexa Fluor 647	BioLegend	135230	250
I-A/I-E (MHCII)	M5/114.15.2	1	Alexa Fluor 700	BioLegend	107621	63
Ly6G	1A8	1	APC-Cy7	BioLegend	127624	20
CD3	17A2	1	APC-Fire810	BioLegend	100268	100

Supplementary Table 2: Final staining list of the 27-color panel (related to Figure 4)

The target specificity of the antibodies, clone, antigen classification. (1=primary, 2=secondary, 3=tertiary), fluorochrome, vendor, catalog number, and dilution used.

**Marker enrichment modeling of neutrophils** (Figure 5d)	
**Cluster**	**Identification**
**1**	$CD44^{-4}$ $Ly6G^{-3}$ $CD24^{-2}$ $CD11c^{+1}$ $CD62L^{+1}$ $PD\text{-}L1^{+1}$ $CD11b^{-1}$ $Ly6C^{+1}$ $CD45^{-1}$
**2**	$CD44^{+9}$ $CD11c^{+6}$ $PD\text{-}L1^{+6}$ $Ly6C^{-5}$ $Ly6G^{-4}$ $CD11b^{-4}$ $CD45^{+4}$ $CD24^{-1}$ $CD62L^{-1}$
**3**	$CD44^{+9}$ $CD11c^{+6}$ $PD\text{-}L1^{+5}$ $Ly6C^{-5}$ $Ly6G^{-4}$ $CD11b^{-4}$ $CD45^{+3}$ $CD24^{-2}$ $CD62L^{-1}$
**4**	$CD44^{+10}$ $Ly6C^{-6}$ $Ly6G^{+2}$ $CD11c^{+1}$ $PD\text{-}L1^{+1}$ $CD45^{+1}$
**5**	$CD44^{+3}$ $Ly6C^{+3}$ $CD24^{-1}$ $Ly6G^{+1}$ $CD45^{+1}$
**6**	$Ly6C^{-5}$ $Ly6G^{-2}$ $CD11c^{+1}$ $CD44^{+1}$ $PD\text{-}L1^{+1}$ $CD45^{+1}$
**7**	$Ly6C^{-5}$ $Ly6G^{+2}$ $CD11c^{+1}$ $CD44^{+1}$ $PD\text{-}L1^{+1}$ $CD45^{+1}$
**8**	$CD44^{-3}$ $Ly6G^{-2}$ $CD11c^{+1}$ $CD62L^{+1}$ $PD\text{-}L1^{+1}$
**9**	$CD11c^{+6}$ $Ly6C^{-5}$ $Ly6G^{-4}$ $PD\text{-}L1^{+4}$ $CD11b^{-4}$ $CD24^{-3}$ $CD45^{+2}$ $CD44^{+1}$
**10**	$CD44^{-4}$ $Ly6G^{-3}$ $CD24^{-2}$ $CD11c^{+1}$ $CD62L^{+1}$ $PD\text{-}L1^{+1}$ $CD45^{-1}$
**11**	$Ly6C^{-5}$ $CD44^{-3}$ $CD11c^{+1}$ $CD62L^{+1}$ $PD\text{-}L1^{+1}$
**12**	$CD44^{-3}$ $CD24^{-1}$ $CD11c^{+1}$ $Ly6G^{-1}$ $CD62L^{+1}$ $PD\text{-}L1^{+1}$
**13**	$CD44^{-3}$ $CD62L^{+1}$ $PD\text{-}L1^{+1}$
**14**	$Ly6C^{-5}$ $CD11c^{+1}$ $Ly6G^{+1}$ $CD62L^{+1}$ $CD44^{-1}$ $PD\text{-}L1^{+1}$
**15**	$Ly6C^{+4}$ $Ly6G^{+1}$ $CD44^{+1}$ $CD45^{+1}$
**16**	$CD44^{+9}$ $Ly6G^{+2}$ $CD11c^{+1}$ $CD45^{+1}$
**17**	$Ly6C^{+10}$ $Ly6G^{+1}$ $CD44^{+1}$ $CD45^{+1}$
**18**	$Ly6G^{+1}$ $CD44^{+1}$ $Ly6C^{+1}$ $CD45^{+1}$
**19**	$CD44^{+9}$ $Ly6G^{+2}$ $CD45^{+2}$
**20**	$CD44^{-3}$ $Ly6G^{-1}$ $CD62L^{+1}$ $PD\text{-}L1^{+1}$ $Ly6C^{+1}$
**21**	$CD44^{+9}$ $CD11c^{+6}$ $PD\text{-}L1^{+6}$ $Ly6C^{-5}$ $Ly6G^{-4}$ $CD11b^{-4}$ $CD45^{+4}$ $CD24^{-2}$ $CD62L^{-1}$
**22**	$Ly6C^{+2}$ $Ly6G^{+1}$ $CD44^{+1}$ $PD\text{-}L1^{+1}$ $CD45^{+1}$
**23**	$Ly6C^{+2}$ $CD62L^{+1}$
**24**	$CD44^{-4}$ $CD24^{-3}$ $Ly6G^{-3}$ $CD11c^{+1}$ $CD62L^{+1}$ $PD\text{-}L1^{+1}$ $CD11b^{-1}$ $Ly6C^{+1}$ $CD45^{-1}$
**25**	$CD44^{+9}$ $CD11c^{+6}$ $PD\text{-}L1^{+6}$ $Ly6C^{-5}$ $Ly6G^{-4}$ $CD11b^{-4}$ $CD45^{+4}$ $CD24^{-2}$ $CD62L^{-1}$
**26**	$Ly6C^{-5}$ $CD11c^{+1}$ $CD62L^{+1}$ $CD44^{-1}$ $PD\text{-}L1^{+1}$ $CD45^{+1}$

Supplementary Table 3: Marker enrichment modeling of neutrophils (related to Figure 5)

Neutrophils (Figure 5) were analyzed for their respective marker enrichments in FlowJo. The MEM creates a value (-10 to +10) for each population feature by quantifying positive and negative population-specific enrichment.

**Marker enrichment modeling of myeloid cells** (Figure 6b)	
**Cluster**	**Identification**
**1**	$CD44^{+5}$ $CD206^{+3}$ $CD11c^{+3}$ $Ly6C^{-3}$ $MHCII^{+1}$ $CD11b^{-1}$ $CD103^{+1}$ $CD24^{+1}$ PD-$L1^{+1}$ $CD64^{+1}$
**2**	$CX3CR1^{+1}$ $CD11b^{+1}$ $CD11c^{+1}$ $CD103^{+1}$ PD-$L1^{+1}$
**3**	$CD206^{-7}$ $CD103^{+1}$ $Ly6C^{+1}$
**4**	$CD206^{-7}$ $CD11b^{+2}$ $Ly6C^{+2}$ $CX3CR1^{+1}$
**5**	$CD206^{+6}$ $CD11b^{+2}$ $CX3CR1^{+1}$ $CD103^{+1}$ $CD64^{+1}$
**6**	$CD206^{+8}$ $CD24^{+5}$ $CD11b^{+2}$ $CX3CR1^{+1}$ $CD64^{+1}$
**7**	$CD206^{+2}$ $CD11b^{+2}$ $MHCII^{+1}$ $CX3CR1^{+1}$ $CD11c^{+1}$ $CD103^{+1}$ $CD44^{+1}$ PD-$L1^{+1}$ $CD64^{+1}$
**8**	$CD11b^{-1}$ $CD103^{+1}$
**9**	$MHCII^{+3}$ $CD24^{+2}$ $CD11b^{-1}$ $CD103^{+1}$ PD-$L1^{+1}$
**10**	$CD206^{-7}$ $Ly6C^{+2}$ $CX3CR1^{+1}$ $CD11b^{+1}$ $CD11c^{+1}$ $CD103^{+1}$ PD-$L1^{+1}$
**11**	$CD206^{-7}$ $CD11b^{+7}$ $Ly6C^{+3}$ $CD64^{+2}$ $CX3CR1^{+1}$ $CD11c^{+1}$ $CD44^{+1}$ PD-$L1^{+1}$
**12**	$MHCII^{+1}$ $CD206^{+1}$ $CD11b^{-1}$ $CD11c^{+1}$ $CD103^{+1}$
**13**	$CD24^{+10}$ $CD206^{-7}$ $MHCII^{+5}$ $CD11c^{+3}$ $CD103^{+2}$ $CD44^{+2}$ $Ly6C^{+2}$ $CX3CR1^{+1}$ $CD11b^{-1}$
**14**	$CD206^{-7}$ $MHCII^{+3}$ $CD24^{+2}$ $CD11b^{-1}$ $CD103^{+1}$ $Ly6C^{+1}$
**15**	$CD206^{-7}$ $MHCII^{+2}$ $CD11b^{+2}$ $Ly6C^{+2}$ $CX3CR1^{+1}$ $CD11c^{+1}$ $CD103^{+1}$ $CD44^{+1}$ PD-$L1^{+1}$ $CD64^{+1}$
**16**	$CD206^{-7}$ $CD24^{+6}$ $Ly6C^{+3}$
**17**	$CD206^{-7}$ $CD24^{+5}$ $CD11b^{+3}$ $Ly6C^{+2}$ $CD44^{+1}$
**18**	$CD206^{-7}$ $MHCII^{+1}$ $CD11b^{-1}$ $CD11c^{+1}$ $CD44^{+1}$ PD-$L1^{+1}$ $Ly6C^{+1}$
**19**	$CD24^{+6}$ $CD11b^{+3}$ $CD44^{+1}$ $Ly6C^{+1}$
**20**	$CD206^{-7}$ $CD24^{+7}$ $CD44^{+4}$ $CD11c^{+3}$ $Ly6C^{+2}$ $MHCII^{+1}$ $CD11b^{-1}$ PD-$L1^{+1}$ $CD64^{+1}$
**21**	$CD206^{-7}$ $CD44^{+6}$ $CD11c^{+3}$ $MHCII^{+1}$ $CD11b^{-1}$ $CD24^{+1}$ PD-$L1^{+1}$ $Ly6C^{+1}$ $CD64^{+1}$
**22**	$CD44^{+5}$ $CD11c^{+3}$ $CD206^{+2}$ $CD11b^{-2}$ $Ly6C^{-2}$ $MHCII^{+1}$ $CD24^{+1}$ PD-$L1^{+1}$ $CD64^{+1}$
**23**	$CD44^{+6}$ $CD11c^{+4}$ $CD206^{+2}$ $CX3CR1^{+1}$ $CD11b^{-1}$ $CD24^{+1}$ PD-$L1^{+1}$ $Ly6C^{-1}$ $CD64^{+1}$
**24**	$CD24^{+8}$ $MHCII^{+7}$ $CD11c^{+2}$ $CD44^{+2}$ $CX3CR1^{+1}$ $CD11b^{-1}$ $CD103^{+1}$ PD-$L1^{+1}$
**25**	$CD24^{+5}$ $CD11b^{+3}$ $CD206^{+1}$ $CD103^{+1}$ $CD44^{+1}$
**26**	$CD24^{+6}$ $CD206^{+4}$ $CD11b^{+2}$ $Ly6C^{+2}$ $CX3CR1^{+1}$
**27**	$CD206^{+4}$ $CD11b^{+2}$ $Ly6C^{+2}$ $CX3CR1^{+1}$ $CD103^{+1}$ $CD64^{+1}$
**28**	$CD206^{+2}$ $CD11b^{+2}$ $Ly6C^{+2}$ $MHCII^{+1}$ $CX3CR1^{+1}$ $CD103^{+1}$ $CD64^{+1}$
**29**	$CD11b^{+3}$ $CX3CR1^{+1}$ $CD206^{+1}$ $CD103^{+1}$ $CD44^{+1}$ $Ly6C^{+1}$ $CD64^{+1}$

Supplementary Table 4: Marker enrichment modeling of myeloid cells (related to Figure 6)

Myeloid cell clusters (Figure 6; non-neutrophils) were analyzed for their respective marker enrichments in FlowJo. The MEM creates a value (-10 to +10) for each population feature by quantifying positive and negative population-specific enrichment.
